# Everybody's Page

**Published:** 1892-01-02

**Authors:** 


### EVERYBODY'S PAGE.

THE use of Caffeine in conjunction with stimulants is advocated by Dr. Gempt, a German physician, in cases of acute pneumonia. Though its action is prompt, he would use it hypodermically in urgent cases.

SOME interesting experiments in the analysis of milk have recently been carried out by Professor A. R. Leeds and Dr. E. P. Davis, of the United States. These experiments have led them to confirm the opinion that sterilised milk, though a useful remedy in various stomach complaints, is insufficient to sustain life. If it is necessary to sterilise milk, the desired effect can be produced without detracting from its digestibility, by first making the milk very slightly alkaline with lime water and then heating to 155 deg. F. for six minutes. By raising the temperature to the boiling point, and retaining it at that point for some time, a considerable portion of the soluble proteids are converted into insoluble. The result of their conclusions is that sterilised milk is less readily and less perfectly digestible than raw milk.

A SOMEWHAT novel argument in support of the multiplication of public-houses, which certainly would scarcely commend itself to Sir Wilfrid Lawson, was advanced by an acquaintance of ours in the course of conversation in a small village not a hundred miles from London. "If," said he, "you have only one public in a village, A, B, and C will meet there of an evening, and in the natural course of events will have a drink. To be sociable A will stand B and C a drink; B and C, not to be mean, will in turn stand drinks, and by degrees all will become hopelessly fuddled, and possibly noisy. But," argued my friend, "if you have several houses in the village, A is sure to prefer one, B another, and C another for some excellent reason. The result is A, B, and C go to their respective haunts, have one drink, and go home peaceful citizens." This argument settles once and for all the question of the public-house. It is quite clear that if we wish to keep our character as sober and moral citizens we must vote against local option, and for an increased number of gin-palaces, of which there is such a dearth in the land!

